# Phosphorus acquisition among planktonic microbial communities across a salinity gradient

**DOI:** 10.1093/ismeco/ycag249

**Published:** 2026-09-02

**Authors:** Thomas Mollica, Elin Lindehoff, Daniel Lundin, Jarone Pinhassi, Malin Olofsson, Kristian Spilling, Hanna Farnelid, Catherine Legrand

**Affiliations:** Linnaeus University, Centre for Ecology and Evolution in Microbial model Systems - EEMiS, SE-39182 Kalmar, Sweden; Interdisciplinary Centre of Marine and Environmental Research (CIIMAR), Terminal de Cruzeiros de Leixões, Av. General Norton de Matos s/n, 4450-208 Matosinhos, Portugal; Linnaeus University, Centre for Ecology and Evolution in Microbial model Systems - EEMiS, SE-39182 Kalmar, Sweden; Linnaeus University, Centre for Ecology and Evolution in Microbial model Systems - EEMiS, SE-39182 Kalmar, Sweden; Linnaeus University, Centre for Ecology and Evolution in Microbial model Systems - EEMiS, SE-39182 Kalmar, Sweden; Department of Aquatic Sciences and Assessment, Swedish University of Agricultural Sciences, Box 7050, 750 07 Uppsala, Sweden; Marine and Freshwater Solutions, Finnish Environment Institute, Latokartanonkaari 11, 00790 Helsinki, Finland; Centre for Coastal Research, University of Agder, Universitetsveien 25, 4604 Kristiansand, Norway; Linnaeus University, Centre for Ecology and Evolution in Microbial model Systems - EEMiS, SE-39182 Kalmar, Sweden; Linnaeus University, Centre for Ecology and Evolution in Microbial model Systems - EEMiS, SE-39182 Kalmar, Sweden; School of Health and Welfare, Jönköping University, SE-55111 Jönköping, Sweden

**Keywords:** Bothnian Bay, Bothnian Sea, phosphorus, stoichiometry, phytoplankton, bacteria, cyanobacteria, metatranscriptome

## Abstract

The Baltic Sea, with its pronounced gradient in temperature, salinity, and nutrient availability, provides a natural system to investigate microbial responses to eutrophication and climate change effects. While nutrient limitation in this system has been extensively studied, direct comparisons of phosphorus acquisition strategies across co-occurring prokaryotic and eukaryotic communities remain limited. This study provides a comprehensive analysis of how microbial plankton communities respond at the molecular level to shifts in phosphorus availability, by resolving both prokaryotic and eukaryotic strategies across spatial and seasonal gradients. By combining size-fractionation, community composition profiling, stoichiometry, and metatranscriptomics along a transect from the Bothnian Bay to the Bothnian Sea across spring and summer conditions, we identify both shared and domain-specific strategies of P acquisition. Results reveal that phosphorus acquisition is shaped by strong environmental gradients, with prokaryotes primarily relying on transporter-mediated uptake, while eukaryotes preferentially employ membrane remodelling and degradation pathways. These contrasting strategies varied across basins and seasons, reflecting differences in nutrient availability, temperature, and community structure. This integrative approach provides new insights into the functional ecology of microbial communities under shifting nutrient regimes and improves our ability to anticipate ecosystem response to eutrophication and climate change in the Baltic Sea.

## Graphical abstract

**Graphical Abstract:**
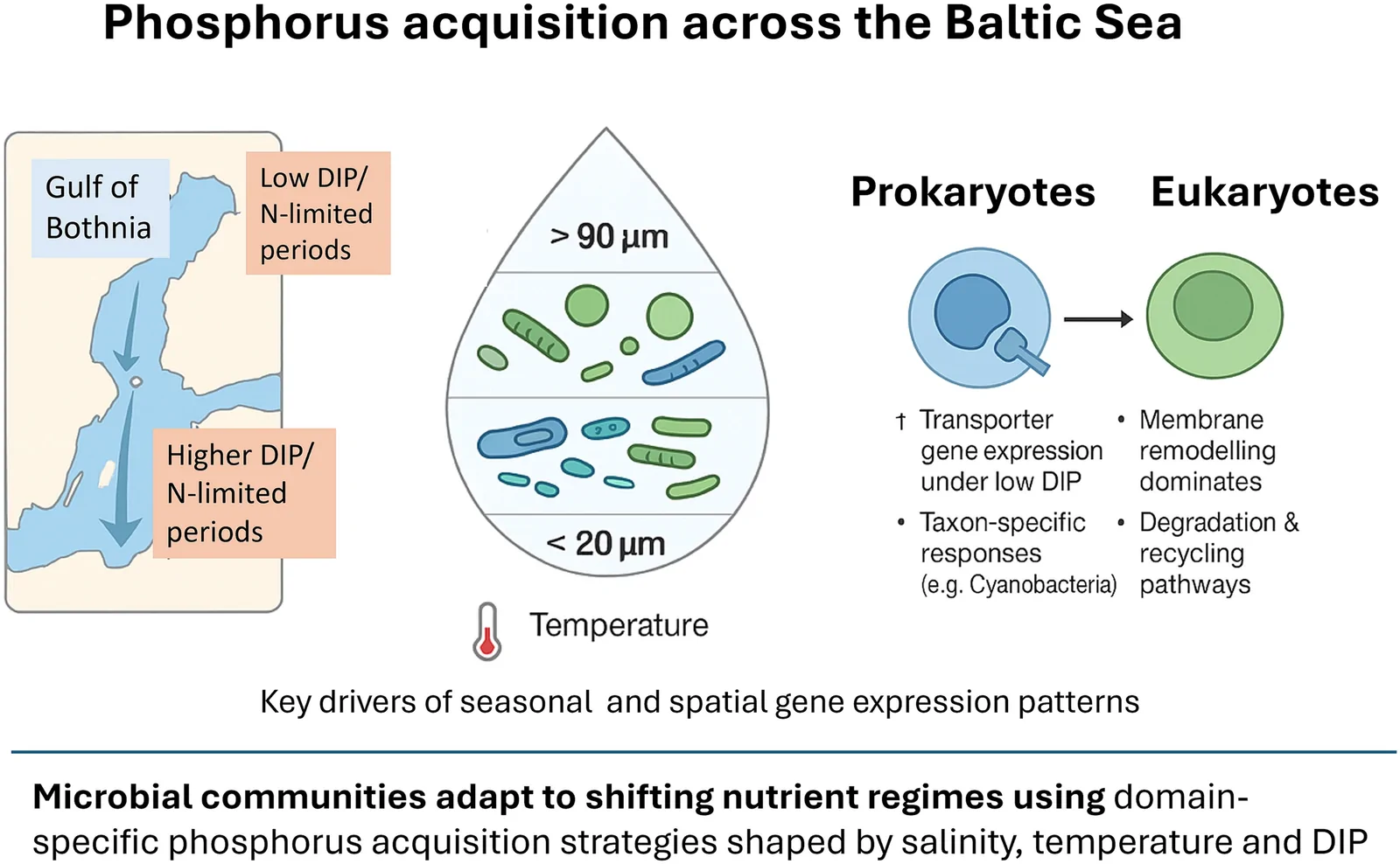

## Introduction

The Baltic Sea, one of the largest brackish water systems on Earth, provides a unique ecosystem due to a distinct North–South gradient in temperature, salinity, and nutrient availability. Its northern region, the Gulf of Bothnia (GoB) provides a unique model system to study eutrophication across a salinity gradient. The GoB comprises two basins, the Bothnian Bay (BoB) and the Bothnian Sea (BoS), with a salinity lower in the BoB (three PSU) compared to the BoS (six PSU), while surface temperatures remain similar [1]. Historically, primary producers in both basins were P-limited [2], but the BoS now experiences seasonal N limitation in spring and summer alongside elevated P concentrations [3].

Following severe eutrophication in the late 20th century [4], the Baltic Marine Environment Protection Commission (HELCOM) initiative implemented nutrient reduction strategies across the Baltic Sea [2]. Between 1997–2003 and 2020, nutrient loads decreased in both basins, but disproportionally: N and P loads decreased similarly in the BoB (−15% and −16%), while the BoS saw a greater reduction in N (−24%) than P (−10%) [5]. This shift has lowered the N:P ratio in the BoS closer to the Redfield ratio (16:1), while the BoB remains P-limited [1, 6]. During spring, long-term monitoring of phytoplankton dynamics in the BoB shows a decline in diatoms and an increase in dinoflagellates and potentially mixotrophic flagellates [1, 7], including nanoflagellates (<20 μm) such as prymnesiophytes, cryptophytes, and prasinophytes [8]. The spring bloom depletes the N pool, leaving an excess of dissolved inorganic phosphorus (DIP) in the system, which is largely converted into particulate organic P (POP) rather than dissolved organic P (DOP) [9]. While picophytoplankton, including picocyanobacteria, assimilate some of this excess P [10], the remaining pool may fuel N_2_-fixing cyanobacterial blooms later in summer. Cyanobacterial biomass has increased in both basins during the last decades with a North–South gradient in species composition; Chroococcales and Oscillatoriales (<3 μm) dominate in the BoB, while Nostocophyceae (~100 μm) dominate in the BoS [8, 11, 12] where the reduced N:P ratio appears to favour their growth [1, 11]. Filamentous N_2_-fixing cyanobacteria can contribute up to 25% of the annual N input, further lowering the DIN:DIP ratio and intensifying P limitation [13–15]. Overall, changes in nutrient and phytoplankton composition will also have cascading effects on bacterial community dynamics and their metabolic strategies. For example, specific diatom species have been shown to influence bacterial community structure in the Baltic Sea [16], and microbial communities may activate enzymes like alkaline phosphatase to scavenge phosphate [17] or employ other strategies such as expressing high affinity P transporters or remodelling phospholipid membranes [18]. Prokaryotes often rely on the PstSCAB complex, under low P conditions [19, 20], while eukaryotes may use equivalent transporters [21, 22], degrade DOP via phosphatases and phosphonases, or recycle internal P though membrane remodelling and polyphosphate utilization [23–25].

On a larger scale, shifts in the balance between dissolved inorganic nitrogen (DIN) and DIP are taking place in different ecosystems. A decrease in the DIN:DIP ratio is typically linked to the deoxygenation of the ocean [26], and the main drivers of this are eutrophication and warming. Warming of the ocean takes place globally and reduces the solubility of oxygen at the surface with implications for the oxygen budget in deeper water masses while eutrophication takes place on local to regional scales, as is the case with the Baltic Sea, where decomposition of the increased organic matter aggravates oxygen consumption at the sea floor. Depletion of oxygen has implications for biogeochemical cycles as microbial respiration shifts to other electron acceptors. For example, in the Baltic Proper under anoxic conditions iron-oxides are being reduced and any sorbed P is being released, which could increase DIP and reduce the DIN: DIP ratio [27, 28].

This study provides a comprehensive analysis of how microbial planktonic communities respond at the molecular level to shifts in P availability by resolving both prokaryotic and eukaryotic strategies across spatial and seasonal gradients. By combining size-fractionation protocols, community composition profiling, stoichiometry, and metatranscriptomics, we identify both shared and domain-specific pathways of P acquisition across the Gulf of Bothnia. This integrative approach provides new insights into the functional ecology of microbial communities under shifting nutrient regimes and improves our ability to anticipate ecosystem responses to eutrophication and climate change.

## Material and methods

### Study area

The samples were collected during three cruises in the BoB and BoS (Fig. 1). The spring cruise (18–28 April 2023) on the R/V Aranda targeted the spring bloom, while the two summer cruises (2–4 August 2022 and 14–16 August 2023) on the KBV 181 were part of the Swedish National Environmental monitoring programme. Water was collected at 2 m depth using a 30 L Ruttner bottle (spring) or a rosette of Niskin bottles (summer).

**Figure 1:**
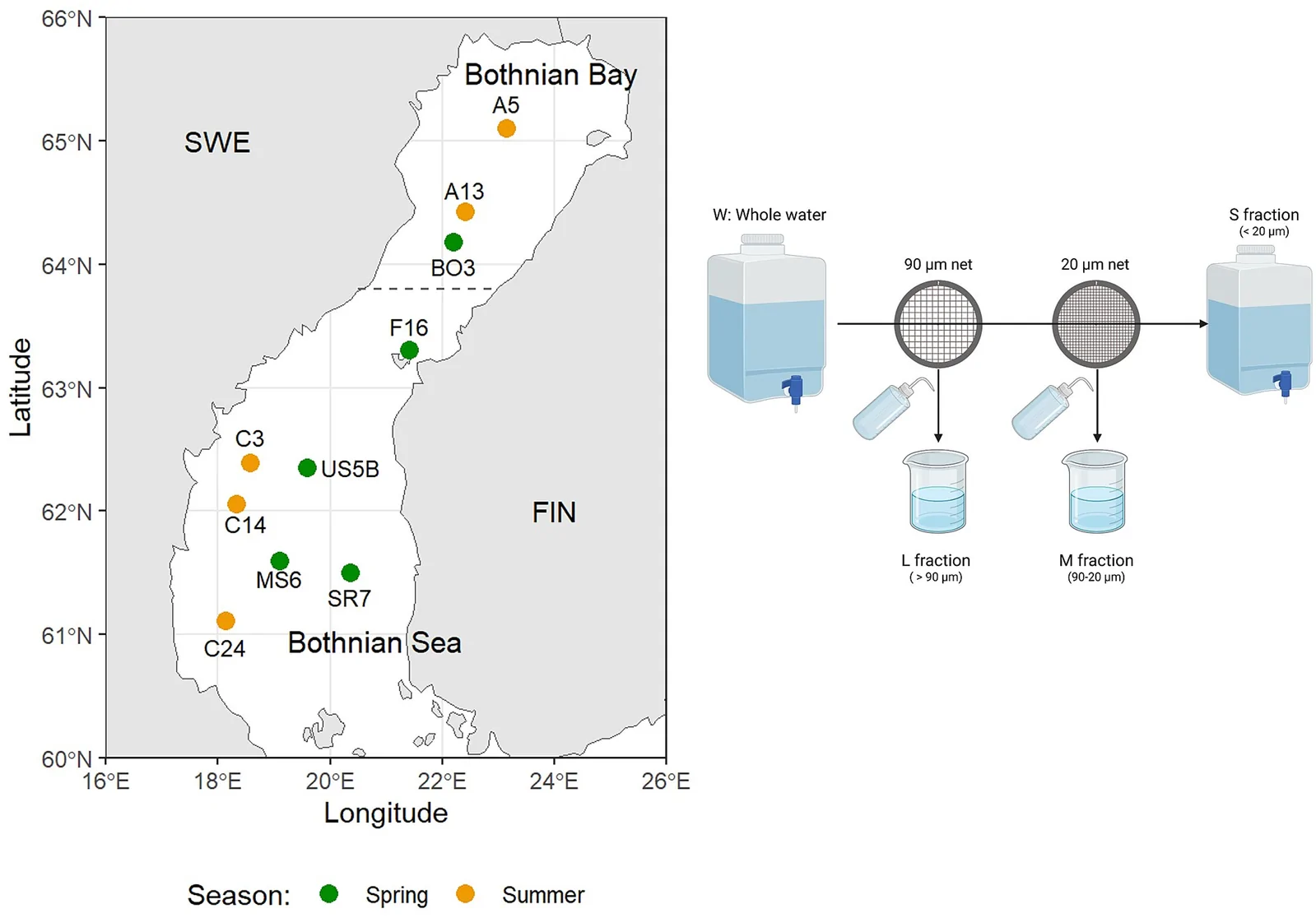
Sampling stations across the Gulf of Bothnia, delineated by a dashed line separating the basins and size fraction protocols employed across the three cruises (Created in BioRender. Mollica, T. (2026) https://BioRender.com/uxvnr0s). The stations sampled were the same for the two summer campaigns.

### Field sampling

The water was size-fractionated into 4 fractions: W (unfiltered water), L (>90 μm), M (20–90 μm), and S (<20 μm, see Fig. 1) fractions. The L fraction aimed to collect filamentous cyanobacteria; the M fraction, large diatoms and dinoflagellates; and the S fraction, nano- and picoplankton. To obtain the L and M fractions, 6 L (summer 2022) or 20 L (spring and summer 2023) of the whole water were subsequently filtered through a 90 and a 20 μm mesh filter (Fig. 1). The biomass collected was resuspended in 250 ml of filtered autoclaved seawater from the Baltic Proper (summer 2022) or in artificial seawater prepared prior to the sampling (spring 2023: 6 PSU, summer 2023: 3 PSU for BoB, 5 PSU for BoS).

To measure chlorophyll *a* concentration as a proxy for biomass, 500 ml of the W fraction were filtered on glass fibre A/E (summer) or GF/F (spring) filters and frozen at −20°C until extraction. Extraction was done with ethanol (summer 4 ml, spring 5 ml) and measured on a Trilogy® Turner Designs (summer) or a Varian Cary Eclipse (Agilent, USA; spring) fluorometer according to Jespersen and Christoffersen [29].

For the summer cruises (2022–2023), all dissolved nutrient data comes from the dBotnia database, collected as part of Swedish National monitoring programme. The dissolved inorganic nutrients were analysed following colorimetric methods [30] and the HELCOM guide [31]. Total nutrient pools were analysed similarly after oxidation using a modified method [30]. During the spring cruise (2023), inorganic nutrients were determined using standard colorimetric methods [30] directly after the samples were taken. For both spring and summer cruises, temperature and salinity were measured using Conductivity, Temperature and Depth (CTD) sensors (SeaBird, USA).

To contextualize field data from the cruises within a yearly framework, environmental data for 2022–2023 was sourced from freely available SHARKweb, the Swedish Marine Archive’s online platform (https://shark.smhi.se/), managed by SMHI. From this dataset, two representative stations each from the BoB (A5 and A13) and the BoS (C3 and C14) were selected for analysis; these were chosen for their comprehensive coverage during the specified period.

### Laboratory analyses

Samples for particulate organic carbon (POC), nitrogen (PON), and phosphorus (POP) were collected on Whatman GF/F filters (pre-combusted at 475°C, 3 h) with a filtered volume of 200–500 ml for W and S fractions and 30 ml for L and M fractions. The filters were kept at −20°C until analysis. Analysis for POC/PON were done on a CHN/S analyser (Perkins-Elmer 2400 Series II), while POP was analysed using a protocol based on Solorzano and Sharp (1980/SS-EN ISO 6878-2005 with modification), absorbance was measured with a Hach Lange DR 3900 fluorometer.

Samples for phytoplankton identification and enumeration were collected by fixation of 100 ml (W and S fractions) or 50 ml (L and M fractions) of water with acid Lugol’s solution and kept in dark at 4°C until analysis. The samples were counted using inverted light microscopy (Nikon TMS, Tokyo, Japan) according to Uthermöhl [32]. Samples for cell enumeration by flow cytometry were taken in triplicates of 1.6 ml of water from fraction S (<20 μm) and fixed with first grade glutaraldehyde at a final concentration of 1%. After a minimum of 5 min incubation, the samples were stored at −20°C until further processing. Measurements were done on a Cytoflex (Beckman Coulter Life Sciences CytoFlex, USA) at a flow rate of 30 μL min^−1^ and analysed with CytExpert (Beckman Coulter Inc). Populations of picocyanobacteria (phycoerythrin and phycocyanin rich), picophytoplankton, and nanophytoplankton were determined based on their pigment content and their size. Size was estimated using forward scatter (FCS-H) as a proxy. Pigments were determined using a blue (488 nm) laser for phycoerythrin (585/42 BP) and chlorophyll *a* (690/50 BP) and a red (638 nm) laser for phycocyanin (660/10 BP). To measure heterotrophic bacteria, cells were stained with SYBR Green I (Thermo Fisher Scientific, Inchinnan, Scotland) to a final concentration of 6× and detected using B525-H (525/40BP) and the side scatter detector (SSC-H).

For both DNA and RNA, triplicates were collected from the W fraction on Sterivex capsule filters (Millipore) with filtered volumes varying between 0.5 and 1 L (DNA and RNA) depending on biomass, volume of available water, and filtration time constraints. RNA samples were filtered for <20 min and 2 ml RNAlater was added to each capsule. During the summer cruises, filters were immediately frozen onboard in liquid N, while for the spring cruise, filters were placed directly at −80°C until extraction.

### DNA extraction, amplification, and sequencing

DNA samples were extracted using FastDNA® SPIN Kit for soil with Lysing matrix Y (MP Biomedicals) according to the manufacturer’s instructions with a supplementary incubation step at 55°C for 1 h with proteinase-K (0.2 μg μL^−1^, 1% final concentration). The V3-V4 region of the 16S rRNA gene was amplified using the 341F and 805R [33] primers (Supplementary Table 1). The V4 region of the 18S rRNA gene was amplified using the primers 454F [34] and V4RB [35] (Supplementary Table 1). Phusion High-Fidelity PCR Master Mix (Thermo Fisher) was used, and the products were purified after each PCR with AMPure XP (Beckman Coulter). For Illumina sequencing, a second PCR was done to attach i5 and i7 NEXTERA indexes using: (1^*^[98°C ^*^30 s], 12^*^[98°C, 10 s, 62°C, 30 s, 72°C, 5 s], 1^*^[72°, 2 min]). The PCR products were quantified with a Qubit fluorometer and pooled at equal concentration. Sequencing was done by SciLifeLab/NGI (Stockholm, Sweden) on Illumina MiSeq 2x300 bp. Gene amplicons of 16S and 18S rRNA were analysed in separate runs through the pipeline nf-core/ampliseq [36] (2.7.0). This Nextflow [37] (22.04.2) implemented pipeline uses DADA2 [38] (1.28.0) to remove sequencing errors from reads and assign taxonomy to the resulting amplicon sequence variants (ASVs). The reference databases used were sbdi-gtdb (R07-RS207-1) [39] for 16S and PR2 (Version 5.0.0) [40] for 18S.

### RNA extraction and sequencing

RNA extraction was done on W fraction samples using Lysing matrix E (MP Biomedicals) and RNeasy mini kit (Qiagen Cat No./ID: 74104). A quality check was done after extraction with Tapestation (Tapestation 4150, Agilent Technologies, USA). The extracted products were treated with DNase using AMBIONTurbo DNA free kit (Invitrogen). Due to low RNA yields, the extracted product was prepared with the Illumina Stranded total RNA Prep (Ribo-Zero Plus) protocol from SciLifeLab/NGI (Stockholm, Sweden) based on reduced rRNA content. The synthesis of complementary DNA (cDNA) and sequencing on Illumina NovaSeq6000 for each sample was done by SciLifeLab/NGI (Stockholm, Sweden).

### Metatranscriptomic analysis

Raw sequence reads were annotated with a *de novo* assembly approach using the pipeline nf-core/metatdenovo (1.0.1) [41] with Megahit (1.2.9) [42] as assembler and KofamScan (1.3.0) [43] for functional annotation. The pipeline was done twice, one run for each kingdom with the following parameters. For the eukaryotic gene expression, Transdecoder (5.7.1) [44] was used to call open reading frames (ORFs), and for the prokaryotic gene expression, Prokka (1.14.6) [45] was used. The taxonomic annotation was done with EUKulele (2.1.2) [46] using the MMETSP (v.2) [47, 48] and GTDB (v.R07-RS207) [49] databases, respectively, for eukaryotic and prokaryotic gene expression. To allow comparison between samples, gene expression was normalized to transcripts per million (tpm).

Genes related to P-acquisition were selected from the metatranscriptomes based on their Kegg ortholog (KO) number and filtered based on their score, e-value, and threshold to have the best gene description for each ORF. The selected genes were categorized into five functional groups: degradation, remodelling, regulation, stock/destock (involved in the formation or degradation of internal P reservoirs), and transporter, with regulation only for the prokaryotic metatranscriptome. A list of the genes used in this study, based on Mollica et al [18], can be found in Supplementary Table 2.

### Equivalent sphere diameter model

An equivalent sphere diameter (ESD) model was used to transform and distribute phytoplankton and picophytoplankton abundances to carbon biomass across the size-fractions. This model was adapted from Mollica (2025) by testing and adjusting it with the enumeration of all size fractions (L, M, and S) from stations BO3, US5B, and SR7 (Fig. 1). Carbon conversion factors per cell originates from HELCOM values [50] (version 2018) and for picoplankton a factor of 243.5 fg C cell was used [51].

### Statistics and plotting

Data handling and plotting were done in R [52] using Tidyverse [53], ggConvexHull [54], cowplot [55], and patchwork [56]. Amplicon libraries were analysed using the phyloseq [57] and SRS [58] packages. Statistical analyses were done using the vegan package [59]. Non-metric dimensional scaling (NMDS) analyses using Bray–Curtis dissimilarities after a Wisconsin double standardization were done to visualize differences in gene expression (tpm) across stations, and PERMANOVA was used to identify the most significant drivers. Correlation tables are based on Spearman correlation with a significance level at ρ = 0.05 and plotted using corrplot [60] and pheatmap [61].

## Results

### Environmental background

Monitoring data showed that in 2022–2023, temperature in the BoB and BoS ranged from 0°C in winter to 15°C in summer (Fig. 2a). Salinity varied between 2.4–3 PSU (BoB) and 4.5–5.6 PSU (BoS), increasing southwards (Fig. 2b). During the spring cruise, temperature followed a North–South gradient with the coldest station (BO3) still ice-covered (Figs 1 and 2a). Phytoplankton biomass differed between basins and seasons. In the BoB, delayed spring and summer blooms due to ice coverage were of comparable magnitude (4.6 and 3.2 μg Chl *a* L^−1^ in 2022 and 2023, respectively). In contrast, the BoS had a pronounced spring bloom (8.5 μg Chl *a* L^−1^), about three times higher than in summer (Fig. 2c). Nitrogen parameters [total nitrogen (TN) and DIN] from SHARKWeb showed similar basin-scale patterns across both years, although DIN was consistently 1.5 times higher in the BoB (Fig. 2d and e). Total phosphorus (TP) was low and constant in the BoB (~0.2 μM) but varied in the BoS, peaking at 0.8 μM after winter. DIP concentrations were lowest in the BoB (0.01–0.12 μM) and highest in the BoS during spring (up to 0.6 μM, Fig. 2f and g).

**Figure 2:**
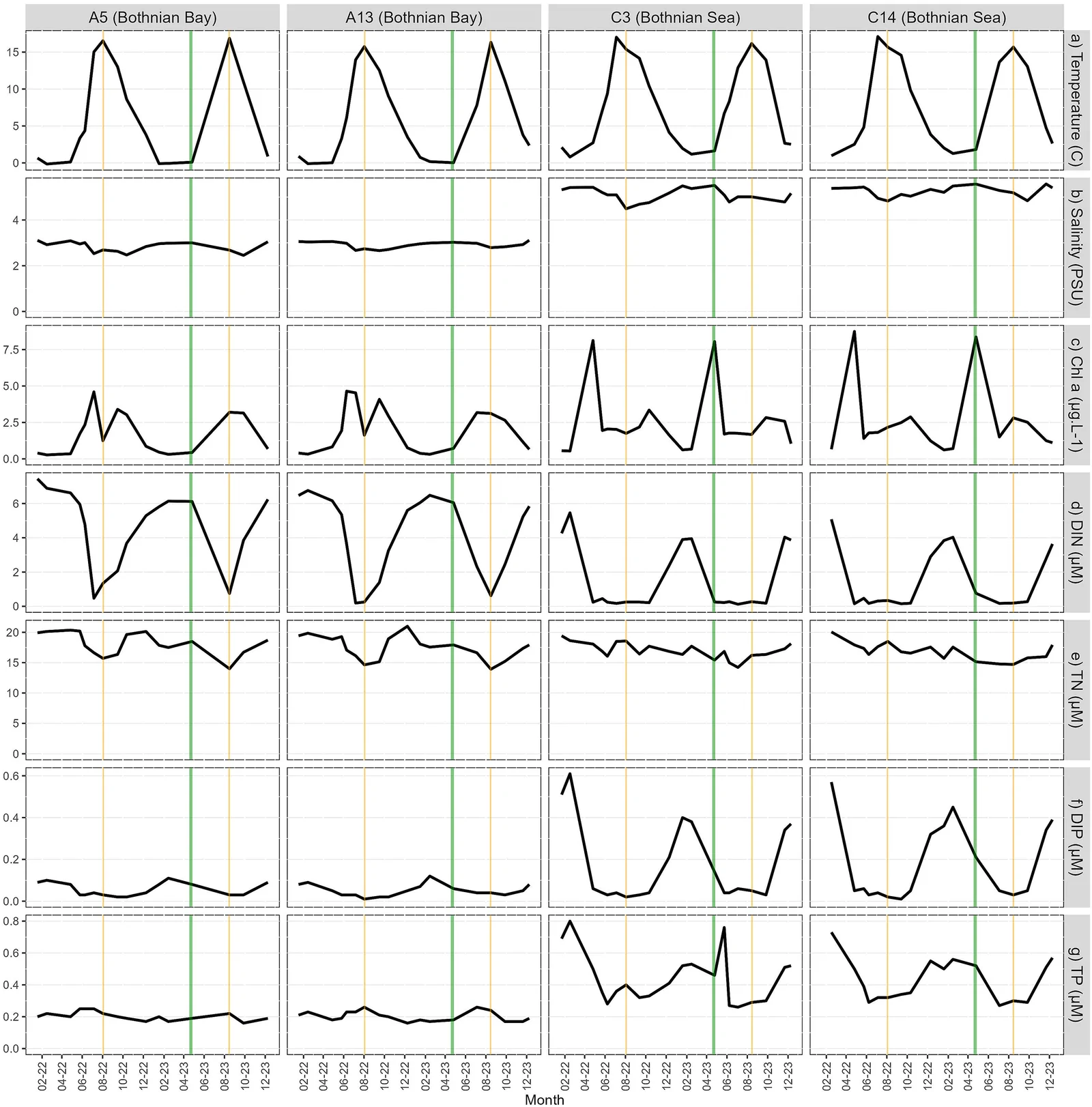
Contextualization of 2022–2023 sampling cruises at two representative stations per basin (from left to right: A5, A13, C3, C14) showcasing a) temperature, b) salinity, c) Chl a, d) DIN, e) TN, f) DIP, and g) TP. Orange bars denote summer cruises, while green bar indicate the spring cruise. Data sourced from SHARKweb database, hosted by SMHI on behalf of the Swedish Agency for Marine and Water Management.

Spring cruise field data revealed a strong North–South gradient in phytoplankton biomass, ranging from 0.84 μg Chl *a* L^−1^ at the ice-covered station BO3 (BoB) to 10.83 μg Chl *a* L^−1^ at station MS6 (BoS). In summer, BoB phytoplankton biomass increased from 2022 to 2023 (1.4 ± 0.2 to 3.0 ± 0.1 μg Chl *a* L^−1^, respectively), while BoS concentrations remained relatively stable (2.1 ± 0.46 and 1.8 ± 0.5 μg Chl *a* L^−1^ in 2022 and 2023, respectively).

### Dissolved nutrient pools and stoichiometry

During the spring cruise, DIN was higher at the northern stations (BO3 and F16; ~5 μM) and depleted in the south (MS6 and SR7; 0.06–0.16 μM; Supplementary Fig. 1) while TN remained relatively constant (~16.8 μM). In summer, DIN was consistently low (<1.4 μM) across all stations, with a similar North–South gradient. TN showed greater variability, with lower levels in the BoB in 2023. DIP increased southwards (0.05–0.22 μM) and TP remained two to three times higher in the BoS than in the BoB. DIP remained low (<0.05 μM) but at least twice as high in 2023 compared to 2022 (Supplementary Fig. 1) while TP remained similar between the two summer cruises.

Stoichiometric ratios (TN:TP and DON:DOP) were consistent across cruises, indicating P as the main limiting nutrient (Fig. 3a). However, DIN:DIP varied significantly. In spring, northern stations were P-limited (DIN:DIP >51; ln = 3.94), while southern stations were strongly N-limited (DIN:DIP = 0.71; ln = −0.33) (Fig. 3a). A similar pattern was observed in summer, with the BoB generally P-limited and the BoS N-limited. Both basins exhibited stronger N-limitation in summer 2023 compared to 2022, most notably at station C14 (BoS, Fig. 1), which shifted from slight P-limitation (DIN:DIP = 17.7; ln = 2.87) to N-limitation (DIN:DIP = 6.3; ln = 1.85) (Fig. 3a).

**Figure 3:**
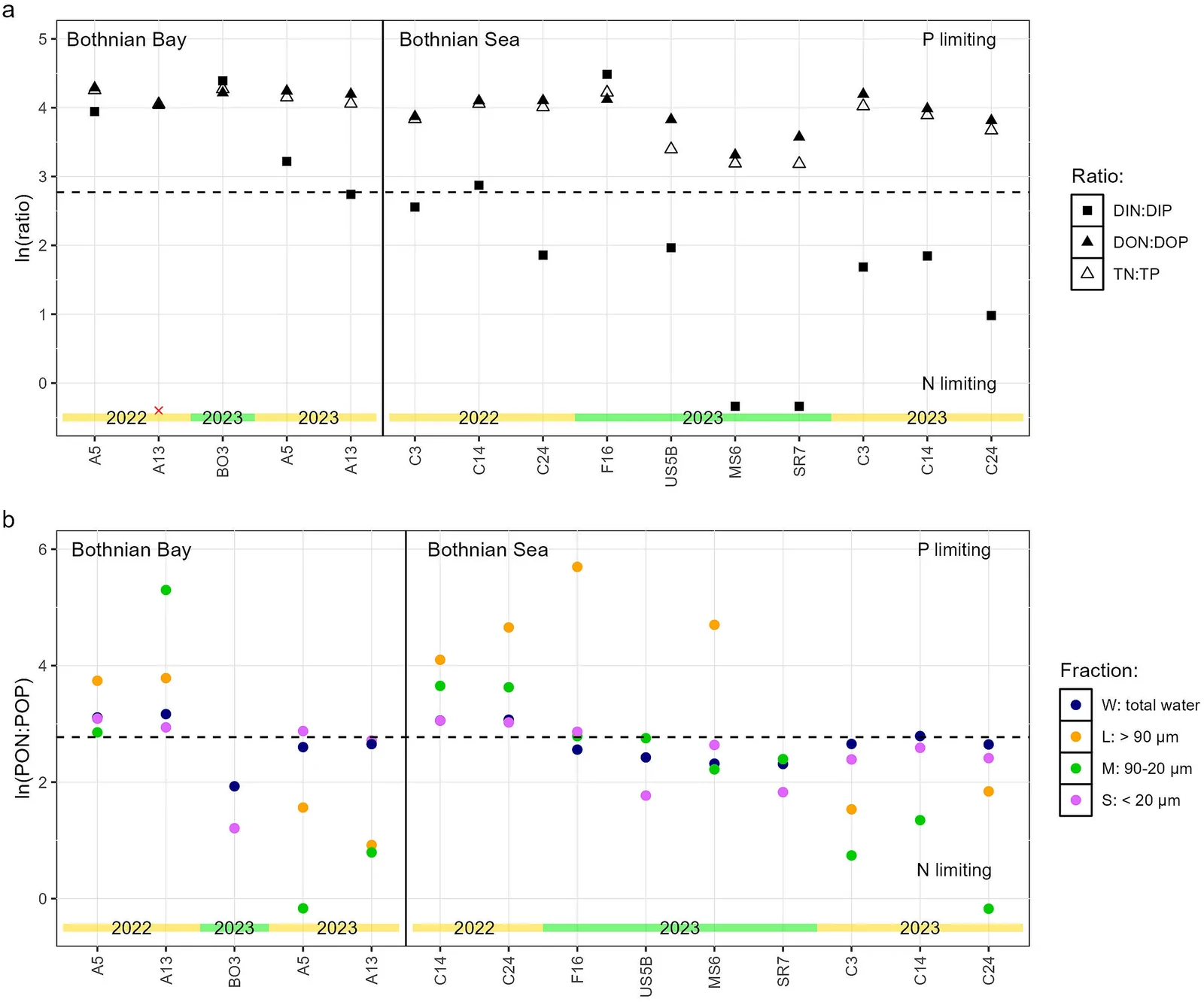
(a) Stoichiometry molar ratios of dissolved nutrient pools for each station, the red cross indicates missing ratios due to phosphorus levels below detection limits for A13. Abbreviations: DIN, dissolved inorganic nitrogen; DON, dissolved organic nitrogen; TN, total nitrogen; DIP, dissolved inorganic phosphorus; DOP, dissolved organic phosphorus; TP, total phosphorus. (b) Seasonal PON:POP molar ratios in planktonic size fractions. For both panels the dashed line represents the Redfield ratio, and sampling cruises are represented with yellow (summer cruises) and green (spring cruise) bars.

### Overview of community biomass and stoichiometry

The S fraction (<20 μm), targeting nanoplankton and picoplankton, dominated seston biomass across all basins and seasons. The S fraction contributed 36%–90% of total biomass across cruises, with the M fraction playing a large role in spring (Supplementary Fig. 2). In summer, the L fraction became more prominent, particularly in the BoB where it accounted for 17% of POC in 2022 and 9% in 2023 (Supplementary Fig. 2). However, estimates for the ESD model indicated that the L fraction contributed only 3% to biomass at station A5 in 2022 and was negligible at other stations (Supplementary Fig. 3). The S fraction also contributed at least 65% of PON and POP in spring, and maintaining high contribution in summer (Supplementary Fig. 2). However, its share of POP declined from 83% in 2022 to 43% in 2023, while the M and L fractions increased their relative contributions. The PON:POP ratio in the W fraction (15.2 ± 5) was comparable to that of the S fraction (14.1 ± 6), both aligning with the Redfield ratio (Fig. 3b). Notably, a shift from P-limitation to N-limitation was observed between summer 2022 and 2023, particularly in the L and M fractions. At station A13 (BoB), the PON:POP ratio in the M fraction dropped from 200 (ln = 5.3) in 2022 to 2.21 (ln = 0.79) in 2023 (Fig. 3b) in 2023. Similarly, the L fraction decreased from 44 (ln = 3.79) to 2.5 (ln = 0.9) over the same period, with similar patterns observed in the BoS (Fig. 3b).

### Overview of community distribution and composition

Phytoplankton biomass, estimated by microscopy, was consistently lower in the BoB than in the BoS across all cruises, averaging 36 μg C L^−1^ in summer and 23 μg C L^−1^ in spring, compared to 259 μg C L^−1^ and 549 μg C L^−1^ in the BoS, respectively (Supplementary Fig. 4). In spring, Diatomophyceae dominated the biomass in both basins, except at station MS6, where Dinophyceae accounted for 54% of the biomass (Fig. 4). In summer, Cyanophyceae, mostly represented by *Aphanizomenon flos-aquae*, dominated the BoS (60%–97%), while the BoB had a more diverse community. Notably, dominant taxa in the BoB shifted between years, where Litostomatea (49%) and Dinophyceae (28%) prevailed in 2022, whereas Prasinophyceae was the major contributor (46%) in 2023. Flow cytometry revealed seasonal shifts in picoplankton populations, with photosynthetic picoeukaryotes being most abundant in spring while picocyanobacteria dominated in summer (Supplementary Table 3).

**Figure 4:**
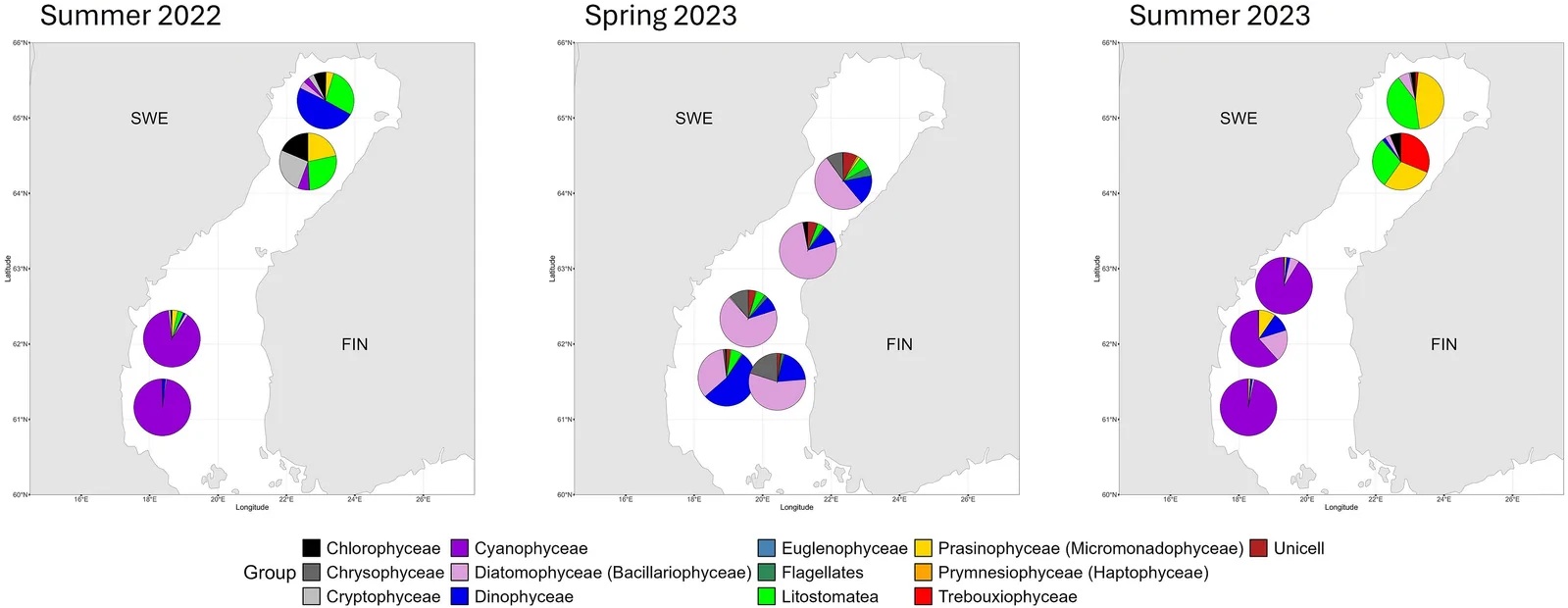
Microscopic enumeration of plankton community distribution and composition in relative carbon biomass for each cruise, with adjusted longitude for BoSS station C3 for improved readability.

Heterotrophic bacteria abundances were generally higher in the BoS, except in summer 2023 when levels were similar across both basins (Supplementary Table 3). The 16S rRNA analysis revealed distinct bacterial community assemblages between cruises. Cyanobacteria were a minor component in the BoB (3%–8%), but more abundant in the BoS, particularly in spring, reaching up to 33% at southern stations MS6 and SR7. A clear North–South gradient in cyanobacterial abundance was observed, supported by microscopy (filamentous and colonial forms), flow cytometry (picocyanobacteria), and 16S rRNA data, with higher relative abundances in spring than in summer (Fig. 5). In contrast, Actinobacteriota, Proteobacteria, and Verrucomicrobiota maintained relatively stable abundances across basins and seasons (30%, 11%, and 6%, respectively). However, Bacteroidota showed more variability, particularly in the BoS in spring ranging from 7% to 32%.

**Figure 5:**
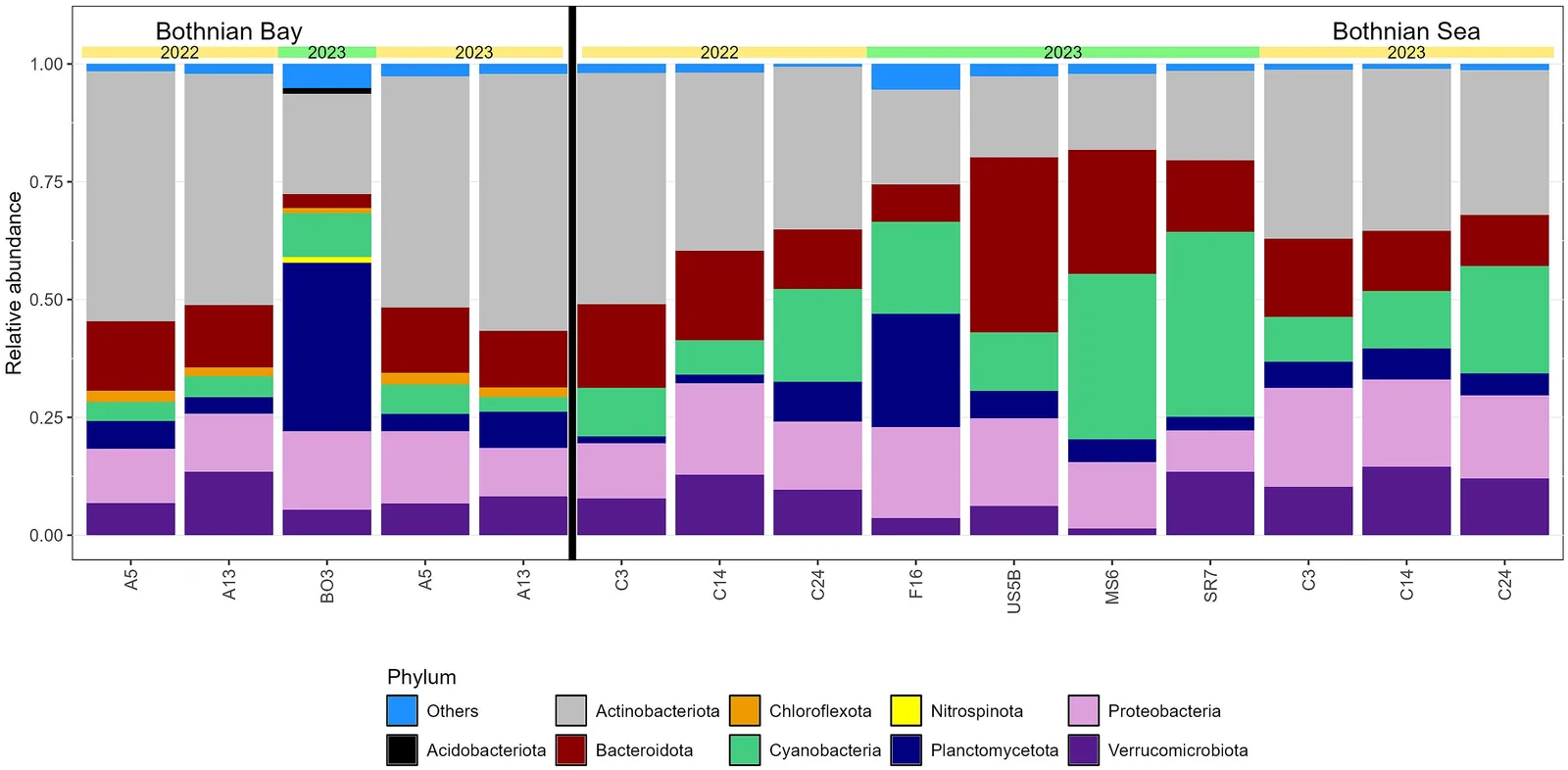
Relative abundance of 16S rRNA gene amplicons in whole water samples across stations along a North–South transect from the Bothnian Bay to the Bothnian Sea. Yellow and green bars representing summer and spring cruises, respectively.

### Diversity of P acquisition strategies in relation to environmental conditions

The P-related gene expression in both prokaryotic and eukaryotic communities was dominated by transporter genes (41.3%), followed by membrane remodelling (33%) and degradation genes (12.3%), with consistent patterns across basins but with seasonal variation (Fig. 6a). Prokaryotes expressed transporter genes at rates 10 times higher than eukaryotes (30%–60%; Fig. 6a, Supplementary Fig. 5), along with regulation and remodelling genes (8%–22%, Supplementary Table 4). During the spring transect, the stock/destock category represented up to 20% of prokaryotic transcripts in spring, but <10% in summer (Supplementary Table 4). NMDS analysis revealed clear seasonal separation in prokaryotic gene expression, with temperature and DIP showing the strongest correlations (*P* < .001, Fig. 6b). Seasonal effect was statistically confirmed (PERMANOVA), with temperature (R^2^ = 0.42, *P* = .001) and chlorophyll *a* (R^2^ = 0.17, *P* = .01) as key drivers. In contrast, eukaryotic communities primarily expressed membrane remodelling genes (61%–74%), followed by degradation (16%–28%) and transporter genes (5%–17%) (Fig. 6a). Stock/destock genes were rarely detected (<1.2%). Seasonal separation was also supported by NMDS, with gene expression correlating most strongly with temperature (*P* = .001) and DIP (*P* < .005), followed by TP, DIN, and Chl *a* (*P* < .05). A negative correlation between temperature and DIP was observed (Fig. 6c). A seasonal effect was observed with temperature explaining the largest variation (PERMANOVA, *adonis*, 999 permutations, R^2^ = 0.31; *P* = .001).

**Figure 6:**
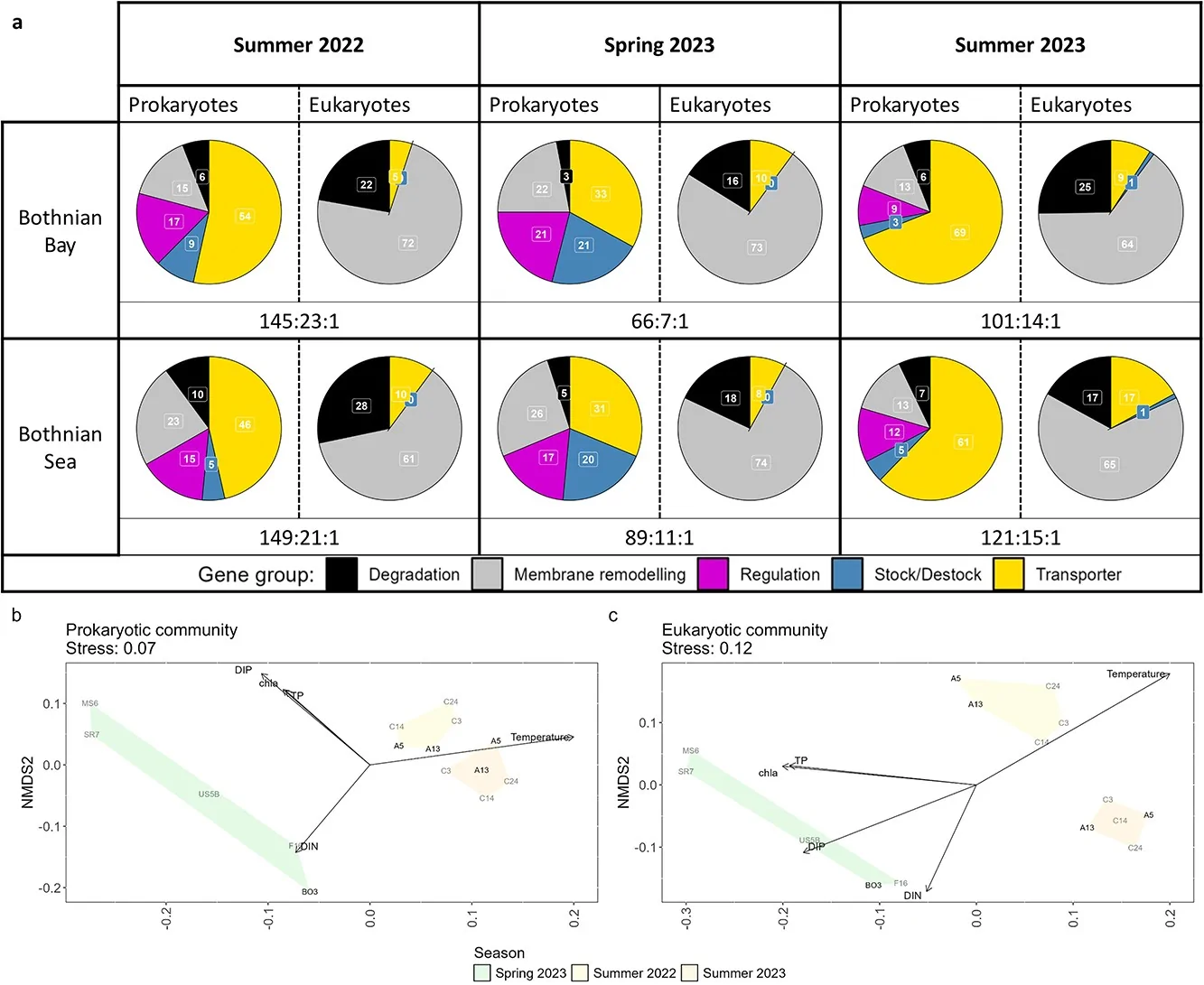
(a) Gene category distribution and relative abundance (in %) in prokaryotic and eukaryotic gene expression across cruises, with average POC:PON:POP ratios per basin (b, c) NMDS plots of community TPM, gene length corrected abundance of transcript in relation to the whole community present in a sample. Per taxonomic class and gene group, with sampling stations coloured by season (coloured panels) and region (black text: Bothnian Bay, grey text: Bothnian Sea). Arrows represent significant environmental vectors (envfit, *P* < .05).

### Dynamics of P acquisition strategies

To assess how P acquisition strategies vary within taxa under different environmental conditions, we calculated the relative expression of P-related genes as a proportion of total transcription within each taxon, referred to as ‘taxon TPM’. Similarly to tpm normalization, which take in account all the transcripts present in one sample, taxon TPM are gene length corrected abundance based on the total number of transcript assigned to one taxon per sample. Taxa were analysed at higher ranks (phylum or class) due to more reliable taxonomic annotation at these levels. Among prokaryotes, Actinobacteria and Cyanobacteria showed the highest TPM values, particularly in the BoB (Fig. 7a). Overall, P-related genes detected in Cyanobacteria were mostly attributed to Synechococcales (72%) and Nostacales (19%). Cyanobacteria and Proteobacteria (alpha, beta, and gamma) showed higher expression in summer compared to spring, while Bacteroidetes remained relatively stable. Gene category distribution varied across taxa and seasons, with Actinobacteria shifting from stock/destock genes in spring to transporters in summer (Fig. 7b). In Alphaproteobacteria, transporters were consistently dominant, though their contribution varied by season and station. Environmental factors influenced gene expression patterns. Temperature positively correlated with transporter gene expression, while DIP correlated negatively with overall P-related gene expression (Supplementary Fig. 6). Stations with high DIP concentrations (US5B and SR7; 0.23 μM) had lower transporter gene expression and higher remodelling activity. In contrast, stations with low DIP (A5 and C14; 0.03 μM) had lower remodelling expression (Supplementary Fig. 6). Betaproteobacteria expressed more regulatory genes at high DIP stations, while Gammaproteobacteria favoured regulation and remodelling genes, with degradation genes more active in summer (Fig. 7b). Salinity was negatively correlated with gene expression in Gammaproteobacteria, particularly in the BoB (Supplementary Fig. 6). Cyanobacteria transporter genes were the most expressed across seasons in the BoB, while remodelling genes peaked in summer 2022 (Fig. 7a). Higher expression of degradation genes was observed at station A13 in summer 2023 compared to 2022, but not at A5, despite similar environmental conditions. In the BoS, regulation gene expression was higher in spring and summer 2023, coinciding with lower seston PON:POP ratios below Redfield (11 and 15), compared to summer 2022 (ratio = 21).

**Figure 7:**
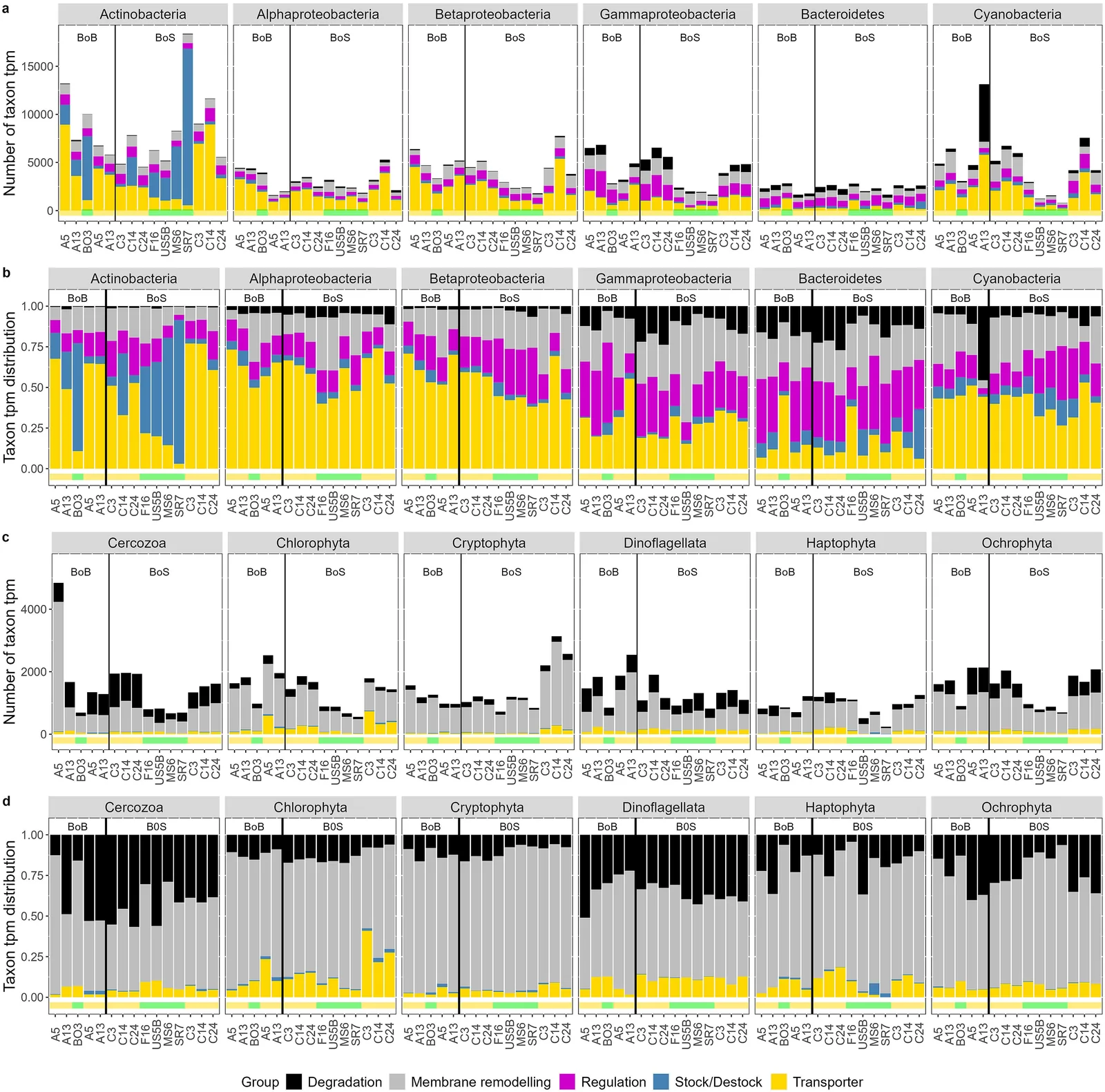
Taxon TPM (gene length corrected abundance normalized to each taxon’s total transcription) and the distribution across main prokaryotic groups (a) and gene categories (b); across main eukaryotic groups (c) and gene categories (d) in the BoB and BoS. Cruises seasons are denoted by horizontal-coloured bars with yellow as summer and green as spring above the station numbers.

Overall, prokaryotic gene expression patterns were strongly influenced by environmental variables (Supplementary Fig. 6). Transporter genes correlated positively with temperature across most taxa, while DIP was the strongest negative predictor of P-related gene expression. Chl *a* was also negatively correlated with remodelling genes across all prokaryote classes and with transporter genes in Alpha- and Betaproteobacteria.

In eukaryotes, taxon-specific gene expression was generally lower compared to prokaryotes and varied between seasons and basins (Fig. 7c). Cercozoa, Chlorophyta, and Ochrophyta showed higher expression in summer, while Dinoflagellata maintained similar levels in the BoS but lower levels in the BoB during spring (Fig. 7c). Across all eukaryotic taxa, membrane remodelling genes were most expressed, followed by degradation genes in Dinoflagellata and transporter genes in Chlorophyta (Fig. 7d). Gene category distribution remained relatively stable across stations and seasons, suggesting taxon-specific strategies regulated by environmental conditions. This was supported by significant correlations between gene expression and temperature and DIP in Chlorophyta and Ochrophyta, while other variables such as salinity and nitrogen showed weak associations (Supplementary Fig. 6).

## Discussion

Phosphorus (P) availability is a central regulator of microbial community structure and function in aquatic ecosystems. In the GoB, seasonal and spatial nutrient gradients, particularly in DIP and N:P ratios, shape microbial strategies for P acquisition and influence biogeochemical processes. Our findings suggest that microbial responses are not solely dictated by nutrient concentrations, but also by bloom dynamics, temperature, and community composition. The shift from P-limitation in 2022 to N-limitation in 2023 in both basins, especially in larger size fractions (L and M), reflects the influence of bloom timing and progression on nutrient stress [62, 63].

Size structure plays a critical role in nutrient cycling. In this study, small plankton (<20 μm) consistently dominated biomass and P-uptake, particularly during spring, aligning with previous studies in the Baltic Sea [51, 64, 65]. However, shifts in stoichiometry and increased contribution from medium (90–20 μm) and large (>90 μm) size fractions suggest that large cells may become more relevant under certain nutrient regimes. The presence of *A. flos-aquae* across all size fractions highlights the complexity of size-based nutrient dynamics. Our analysis of *A. flos aquae* filament structure revealed a discrepancy in its expected size fraction distribution. While its cell morphology suggested presence in the small fraction, filament length (>90 μm) suggested dominance in the large fraction. Surprisingly, our corrected ESD model showed that *Aphanizomenon* was the dominant biomass component across all fractions during summer in the BoS. This may be due to short filaments or breakage of longer filaments during fractionation, supported by molecular identification in both size fractions. These findings suggest that *Aphanizomenon* may be a primary consumer of P in the BoS, potentially sustaining prolonged N₂ fixation and biomass growth. This is supported by the transcript profile of Nostocales-like where transporter-related genes were the most expressed, especially genes coding for the high-affinity transporter complex PstSCAB. This could have cascading effects on food web quality, as cyanobacteria are considered low-quality food for zooplankton [66]. Understanding these dynamics is essential for predicting nutrient fluxes and feedback on primary production under changing environmental conditions as larger fractions may play a greater role in P retention during summer, while smaller fractions drive uptake and cycling during periods of low P availability.

Microbial communities in the GoB exhibit diverse strategies for coping with P-limitation, shaped by their physiological traits and environmental conditions, prokaryotes predominantly rely on high-affinity transporter genes, enabling rapid uptake and metabolic flexibility under low P-conditions [19, 20]. In contrast, eukaryotes favour membrane remodelling and degradation pathways, consistent with structural and biochemical adaptations to P stress [23, 67]. These differences reflect fundamental physiological constraints, such as the inability of eukaryotes to bypass N requirements in membrane synthesis [23].

Within prokaryotes, two dominant acquisition profiles emerged: transporter gene expression in Cyanobacteria and Alpha-/Betaproteobacteria, and regulation/remodelling gene expression in Bacteroidetes and Gammaproteobacteria. These patterns were consistent across basins and seasons, with Actinobacteria showing seasonal shifts (stock/destock genes dominating in the spring and transporter genes in summer). Notably, membrane remodelling genes were widespread across prokaryotic communities in both the BoB and BoS. Phospholipase C (*plc*), a key enzyme involved in phospholipid substitution under P-limitation, was expressed at low levels in Actinobacteria, Bacteroidetes, and Gammaproteobacteria [24, 68]. This suggests that while remodelling is a common strategy, its genetic basis may vary across taxa or be underrepresented in environmental data.

The dominance of sulfolipid pathways (SQD1/SQD2) in Cyanobacteria and widespread expression of remodelling genes across bacterial taxa point to conserved strategies for economizing P under stress [18, 23, 69]). The unexpectedly high gene expression in summer 2023, despite less pronounced P-limitation as indicated by stoichiometry, may reflect increased N₂ fixation, as already observed among filamentous cyanobacteria [70]. Our findings highlight the underestimated role of picocyanobacteria in nutrient cycling, with Synechococcales-like taxa accounting for most of the cyanobacterial gene expression.

Eukaryotic taxa showed taxon-specific P acquisition strategies. Chlorophyta and Ochrophyta relied heavily on membrane remodelling, especially in summer, suggesting active adaptation to low DIP [67]. Dinoflagellata expressed more degradation genes, potentially enabling scavenging of organic P from detrital or dissolved organic matter [71]. The absence of polyphosphate-related genes (e.g. VTC complex) [72–74] may reflect limitations in current environmental gene annotation rather than true absence, highlighting the need of improved functional characterization of eukaryotic P metabolism.

Gene expression patterns in microbial communities across the salinity gradient were strongly shaped by environmental factors, particularly DIP, temperature, and N:P stoichiometry. DIP emerged as the most consistent driver, scaling inversely with P-related genes across taxa. Temperature positively correlated with transport gene expression, while chlorophyll *a* was negatively associated with remodelling and transporter genes in several bacterial classes. Salinity effects were more taxon-specific, notably influencing Gammaproteobacteria [24, 68]. Notably, P-transporter gene expression was approximately 10 times higher in prokaryotes than in eukaryotes, indicating their efficiency in low-P conditions and their capacity for rapid transcriptional response. This expression increased significantly during summer, likely reflecting elevated metabolic activity and nutrient demand. In contrast, eukaryotic phytoplankton exhibited seasonal shifts in gene expression patterns similar to Arctic marine protists [75]. Stock/destock genes contributed more during spring (~20%), suggesting a strategy of seasonal internal P storage, while summer conditions with lower P availability triggered a shift towards active uptake mechanisms.

These findings highlight the functional divergence between prokaryotic and eukaryotic communities in their response to nutrient stress. Prokaryotes demonstrate high ability through efficient uptake systems, while eukaryotes rely more on structural and biochemical adjustments to maintain cellular function under fluctuating environmental conditions.

### Implications and future directions

This study demonstrates that microbial P acquisition strategies in the Gulf of Bothnia are structured by the interplay between environmental gradients, bloom dynamics, and community composition. By integrating prokaryotic and eukaryotic responses within the same environmental framework, we show that similar nutrient conditions can give rise to fundamentally different molecular strategies across domains, highlighting a functional divergence that is not captured in single-community analyses. Importantly, our results indicate that P acquisition is not solely governed by stoichiometric constraints, but also strongly shaped by temperature, community structure, and seasonal dynamics across basins. This underscores the need to consider both environmental forcing and biological organization when interpreting nutrient limitation in aquatic systems. As climate change and eutrophication continue to alter nutrient regimes in the Baltic Sea, a mechanistic understanding of microbial nutrient acquisition strategies will be critical to predict ecosystem responses. The integrative framework presented here provides a basis for linking molecular processes to ecosystem-scale dynamics and highlights the importance of cross-domain approaches for linking molecular processes to ecosystem-level biogeochemical cycling.

## Supplementary Material

Supplementary_material_revision_ycag249_TC_off

## Data Availability

The metabarcoding data used in this study have been deposited in GenBank under the accession number PRJNA1238810. The metatranscriptomic data used in this study have been deposited in GenBank under the accession number PRJNA1311320.
