# Supplementary material for: Phosphorus acquisition among planktonic microbial communities across a salinity gradient
Source: ISME Commun. 2026 Sep 2;6(1):ycag249. doi: 10.1093/ismeco/ycag249 (PMC13617579; doi:10.1093/ismeco/ycag249)
Supplement: Supplementary file 1 [file supplementary_material_revision_ycag249_tc_off.docx]

# Supplementary figures

*Supplementary Figure 1: Nutrient pools for all stations. The yellow bars represent the summer cruises, the green bars the spring cruise. DIN: dissolved inorganic nitrogen, DON: dissolved organic nitrogen, TN: total nitrogen; DIP: dissolved inorganic phosphorus, DOP: dissolved organic phosphorus, TP: total phosphorus*

*Supplementary Figure 2: Distribution of particulate organic carbon (POC), nitrogen (PON) and phosphorus (POP) across the fractions. The yellow bars represent the summer cruises, the green bars the spring cruise.*

*Supplementary Figure 3: Contribution of total carbon biomass of each fraction. Longitude of station C3 for BoSS has been modified for better readability.*

*Supplementary Figure 4: Total phytoplankton carbon biomass estimated by microscopy. The yellow bars represent the summer cruises, the green bars the spring cruise.*

*Supplementary Figure 5:.Number of community TPMs detected for each transcriptome data set for each basin and season. The yellow bars represent the summer cruises, the green one the spring cruise.*

*Supplementary Figure SEQ Supplementary_Figure \* ARABIC 6: Correlations between environmental parameters and each gene category for the main cyanobacteria genera and phytoplankton classes. Each value represents a significant Spearman correlation (p-value < 0.05) and blank square to a non-significant value. DIP:dissolved inorganic phosphorus, DOP: dissolved organic phosphorus, TP: total phosphorus, DIN:dissolved inorganic nitrogen, DON: dissolved organic nitrogen, TN: total nitrogen*

*Supplementary Figure 6*: *Spearman correlation matrix for the prokaryotic and eukaryotic communities metatranscriptomic profiles, blank square indicates insignificant correlations*.

*Supplementary Table 1: Details PCR protocols*

|  | PCR protocol | PCR 2 protocol |
| --- | --- | --- |
| 16S | (1*[98⁰C *30s], 20*[98⁰C, 10s; 58⁰C, 30s; 72⁰C, 15s], 1*[72⁰, 2 min]) | (1*[98⁰C *30s], 12*[98⁰C, 10s; 62⁰C, 30s; 72⁰C, 5s], 1*[72⁰, 2 min]) |
| 18S | (1*[98⁰C *30s], 20*[98⁰C, 10s; 53⁰C, 30s; 72⁰C, 15s], 1*[72⁰, 2 min]) | 1*[98⁰C *30s], 12*[98⁰C, 10s; 62⁰C, 30s; 72⁰C, 5s], 1*[72⁰, 2 min]) |

*Supplementary Table 2: List of the genes analysed in this study. E.C. numbers have been added for membrane remodelling when available.*

| Category | ko | Gene name | Enzyme Commission (EC) number | Function |
| --- | --- | --- | --- | --- |
| Degradation | K19670 | *phnA* |  | Hydrolase |
|  | K05306 | *phnX* |  |  |
|  | K06163 | *phnJ* |  | Lyase |
|  | K14379 | *ACP5* |  | Phosphatase |
|  | K22390 | *ACP7* |  |  |
|  | K06162 | *phnM* |  |  |
|  | K01077 | *pho** |  |  |
|  | K01113 | *phoD* |  |  |
|  | K09474 | *phoN* |  |  |
|  | K06167 | *phnP* |  | Phosphodiesterase |
|  | K02043 | *phnF* |  | Phosphonate transport |
|  | K05781 | *phnK* |  |  |
|  | K03430 | *phnW* |  | Transaminase |
|  | K06166 | *phnG* |  | Transferase |
|  | K06165 | *phnH* |  |  |
|  | K06164 | *phnI* |  |  |
|  | K05780 | *phnL* |  |  |
| Remodelling | K13621 | *BTA1* |  | Betaine lipid synthase |
|  | K01521 | *cdh* | 3.6.1.26 | Hydrolase |
|  | K06124 | *PHOSPHO1* | 3.1.3.75 |  |
|  | K00111 | *glpA/glpD* | [1.1.5.3](https://www.genome.jp/entry/1.1.5.3) | Oxidoreductase |
|  | K00112 | *glpB* | [1.1.5.3](https://www.genome.jp/entry/1.1.5.3) |  |
|  | K00113 | *glpC* |  |  |
|  | K00006 | *GPD1* | 1.1.1.8 |  |
|  | K00057 | *gpsA* | 1.1.1.94 |  |
|  | K18693 | *DPP1, DPPL, PLPP4_5* | 3.6.1.75 ; 3.1.3.4 | Phosphatase |
|  | K15728 | *LPIN* | [3.1.3.4](https://www.genome.jp/entry/3.1.3.4) |  |
|  | K01080 | *PLPP1_2_3* | [3.1.3.4](https://www.genome.jp/entry/3.1.3.4) |  |
|  | K01004 | *pcs* | [2.7.8.24](https://www.genome.jp/entry/2.7.8.24) | Phosphatidylcholine synthase |
|  | K08729 | *PTDSS1* | 2.7.8.- | Phosphatidylserine synthase |
|  | K08730 | *PTDSS2* | [2.7.8.29](https://www.genome.jp/entry/2.7.8.29) |  |
|  | K01126 | *glpQ, ugpQ* | [3.1.4.46](https://www.genome.jp/entry/3.1.4.46) | Phosphodiesterase |
|  | K06900 | *capV* | [3.1.1.32 ; 3.1.1.-](https://www.genome.jp/entry/3.1.1.32) | Phospholipase |
|  | K16818 | *DAD1* | [3.1.1.32](https://www.genome.jp/entry/3.1.1.32) |  |
|  | K22389 | *LCAT3* | [3.1.1.32](https://www.genome.jp/entry/3.1.1.32) |  |
|  | K01047 | *PLA2G, SPLA2* | [3.1.1.4](https://www.genome.jp/entry/3.1.1.4) |  |
|  | K16817 | *PLA2G16* | 3.1.1.32 ; 3.1.1.4 |  |
|  | K16342 | *PLA2G4, CPLA2* | [3.1.1.4](https://www.genome.jp/entry/3.1.1.4) |  |
|  | K16343 | *PLA2G6, IPLA2* | [3.1.1.4](https://www.genome.jp/entry/3.1.1.4) |  |
|  | K14621 | *PLB1, PLB* | 3.1.1.4 ; 3.1.1.5 |  |
|  | K01114 | *plc* | [3.1.4.3](https://www.genome.jp/entry/3.1.4.3) |  |
|  | K17717 | *pld* | [3.1.4.4](https://www.genome.jp/entry/3.1.4.4) |  |
|  | K01115 | *PLD1_2* | [3.1.4.4](https://www.genome.jp/entry/3.1.4.4) |  |
|  | K16860 | *PLD3_4* | [3.1.4.4](https://www.genome.jp/entry/3.1.4.4) |  |
|  | K01058 | *pldA* | 3.1.1.32 ; 3.1.1.4 |  |
|  | K22697 | *SAMD8* | [3.1.4.62](https://www.genome.jp/entry/3.1.4.62) |  |
|  | K04714 | *SGMS* | 2.7.8.27 ; 3.1.4.62 |  |
|  | K14674 | *TGL4* | 3.1.1.3 3.1.1.13 3.1.1.4 2.3.1.51 |  |
|  | K14286 | *AGXT2L1, ETNPPL* | [4.2.3.2](https://www.genome.jp/entry/4.2.3.2) | Phospho-lyase |
|  | K13523 | *AGPAT3_4* | [2.3.1.51 ; 2.3.1.-](https://www.genome.jp/entry/2.3.1.51) | Transferase |
|  | K19007 | *AGPAT5* | [2.3.1.51](https://www.genome.jp/entry/2.3.1.51) |  |
|  | K13509 | *AGPATa_2* | [2.3.1.51](https://www.genome.jp/entry/2.3.1.51) |  |
|  | K13622 | *btaA* |  |  |
|  | K13623 | *btaB* |  |  |
|  | K00981 | *CDS1, CDS2, cdsA* | [2.7.7.41](https://www.genome.jp/entry/2.7.7.41) |  |
|  | K13644 | *CEPT1* | 2.7.8.1 ; 2.7.8.2 |  |
|  | K14156 | *CHK* | 2.7.1.32 ; 2.7.1.82 |  |
|  | K00994 | *CHPT1, CPT1* | 2.7.8.2 |  |
|  | K00866 | *CKI1* | [2.7.1.32](https://www.genome.jp/entry/2.7.1.32) |  |
|  | K07029 | *dagK* | [2.7.1.107](https://www.genome.jp/entry/2.7.1.107) |  |
|  | K16368 | *DGK1* | [2.7.1.174](https://www.genome.jp/entry/2.7.1.174) |  |
|  | K00901 | *dgkA, DGK* | [2.7.1.107](https://www.genome.jp/entry/2.7.1.107) |  |
|  | K00894 | *ETNK, EKI* | [2.7.1.82](https://www.genome.jp/entry/2.7.1.82) |  |
|  | K13507 | *GAT* | 2.3.1.15 ; 2.3.1.42 |  |
|  | K00649 | *GNPAT* | [2.3.1.42](https://www.genome.jp/entry/2.3.1.42) |  |
|  | K13513 | *LCLAT1, AGPAT8* | [2.3.1.- ; 2.3.1.51](https://www.genome.jp/entry/2.3.1.51) |  |
|  | K22831 | *LOA1* | [2.3.1.51](https://www.genome.jp/entry/2.3.1.51) |  |
|  | K13510 | *LPCAT1_2* | 2.3.1.23 ; 2.3.1.67 |  |
|  | K13515 | *LPCAT3, MBOAT5* | 2.3.1.23 ; 2.3.1.- |  |
|  | K13512 | *LPCAT4, AGPAT7* | [2.3.1.23 ; 2.3.1.-](https://www.genome.jp/entry/2.3.1.23) |  |
|  | K13519 | *LPT1, ALE1* | 2.3.1.51 ; 2.3.1.23 ; 2.3.1.- |  |
|  | K13517 | *MBOAT1_2* | [2.3.1.51 ; 2.3.1.-](https://www.genome.jp/entry/2.3.1.51) |  |
|  | K05929 | *NMT* | [2.1.1.103](https://www.genome.jp/entry/2.1.1.103) |  |
|  | K00968 | *PCYT1* | [2.7.7.15](https://www.genome.jp/entry/2.7.7.15) |  |
|  | K00967 | *PCYT2* | [2.7.7.14](https://www.genome.jp/entry/2.7.7.14) |  |
|  | K00551 | *PEMT* | 2.1.1.17 ; 2.1.1.71 |  |
|  | K00550 | *PLMT* | [2.1.1.71](https://www.genome.jp/entry/2.1.1.71) |  |
|  | K00655 | *plsC* | [2.3.1.51](https://www.genome.jp/entry/2.3.1.51) |  |
|  | K00570 | *pmtA* | 2.1.1.17 ; 2.1.1.71 |  |
|  | K06119 | *SQD2* | 2.4.1.- |  |
|  | K06118 | *SQD1* | [3.13.1.1](https://www.genome.jp/entry/3.13.1.1) | UDP-sulfoquinovose synthase |
| Regulation | K07657 | *phoB* |  | Signal Transduction |
|  | K07658 | *phoB1, phoP* |  |  |
|  | K06217 | *phoH, phoL* |  |  |
|  | K07175 | *phoH2* |  |  |
|  | K07636 | *phoR* |  |  |
|  | K07776 | *regX3* |  |  |
|  | K07768 | *senX3* |  |  |
|  | K02039 | *phoU* |  | Transporter |
| Stock/Destock | K01524 | *ppx-gppA* |  | Exopolyphosphatase |
|  | K00937 | *ppk1* |  | Polyphosphate kinase |
|  | K22468 | *ppk2* |  |  |
|  | K23753 | *ppk2, pap* |  |  |
| Transporter | K02041 | *phnC* |  | Transporter |
|  | K02044 | *phnD* |  |  |
|  | K02042 | *phnE* |  |  |
|  | K08176 | *PHO84* |  |  |
|  | K14430 | *PHO87_91* |  |  |
|  | K16322 | *pit* |  |  |
|  | K03306 | *PiT** |  |  |
|  | K02038 | *pstA* |  |  |
|  | K02036 | *pstB* |  |  |
|  | K02037 | *pstC* |  |  |

*Supplementary Table 3: Flow cytometry data for each station sampled at each cruise. PE-rich: phycoerythrin-rich picocyanobacteria, PC-rich: phycocyanin-rich cyanobacteria. PPE: phytopicoeukaryotes*

| **Basin** | **Period** | **Station** | **Total events (autofluorescence)** | **PE-rich (%)** | **PC-rich (%)** | **PPE (%)** | **Nanoflagellates (%)** | **Bacteria (µL-1)** |
| --- | --- | --- | --- | --- | --- | --- | --- | --- |
| Bothnian Bay | Summer 2022 | A5 | 89790 | 70.34 | 28.81 | 0.85 | 0 | 1543.66 |
|  |  | A13 | 191500 | 98.37 | 0.79 | 0.84 | 0 | 1791.67 |
|  | Spring 2023 | BO3 | 13085 | 59.38 | 0 | 40.62 | 0 | 623.49 |
|  | Summer 2023 | A5 | 90240 | 88.34 | 3.7 | 6.41 | 1.56 | 1164.02 |
|  |  | A13 | 122335 | 94.13 | 0 | 4.62 | 1.25 | 1693.76 |
| Bothnian Sea | Summer 2022 | C14 | 340150 | 99.13 | 0.26 | 0.61 | 0 | 2399.17 |
|  |  | C24 | 379820 | 97.85 | 1.34 | 0.81 | 0 | 2402.37 |
|  | Spring 2023 | F16 | 18985 | 45.69 | 0 | 54.31 | 0 | 714.65 |
|  |  | US5B | 25400 | 47.13 | 0 | 52.87 | 0 | 1257.53 |
|  |  | MS6 | 47910 | 45.47 | 0 | 54.53 | 0 | 1119.47 |
|  |  | SR7 | 51450 | 63.07 | 0 | 36.93 | 0 | 1214.65 |
|  | Summer 2023 | C3 | 184760 | 96.56 | 0 | 2.92 | 0.53 | 1388.03 |
|  |  | C14 | 272295 | 94.04 | 0 | 5.56 | 0.4 | 1553.38 |
|  |  | C24 | 127430 | 79.76 | 0 | 19.41 | 0.82 | 1686.75 |

*Supplementary table 4: Total community TPM per group (bacteria/eukaryotes) and relative distribution per gene class for each sampling campaign*

| Basin | Domain | Year | Season | Degradation | Regulation | Membrane remodelling | Stock/destock | Transporter | Total tpm |
| --- | --- | --- | --- | --- | --- | --- | --- | --- | --- |
| Bothnian Bay | Bacteria | 2022 | Summer | 5.6% | 16.96% | 14.56% | 8.75% | 54.13% | 484.95 |
|  |  | 2023 | Spring | 3.14% | 21.29% | 21.53% | 20.79% | 33.24% | 586.02 |
|  |  | 2023 | Summer | 6% | 8.84% | 12.78% | 3.44% | 68.94% | 728.79 |
|  | Eukaryota | 2022 | Summer | 22.31% |  | 72.49% | 0.4% | 4.8% | 240.26 |
|  |  | 2023 | Spring | 16.35% |  | 73.33% | 0.32% | 10.01% | 345.17 |
|  |  | 2023 | Summer | 25.11% |  | 64.35% | 1.13% | 9.41% | 402.04 |
| Bothnian Sea | Bacteria | 2022 | Summer | 10.42% | 15.34% | 22.93% | 5.21% | 46.11% | 449.99 |
|  |  | 2023 | Spring | 5.09% | 17.46% | 26.07% | 20.35% | 31.03% | 244.06 |
|  |  | 2023 | Summer | 7.09% | 12.5% | 13.46% | 5.48% | 61.48% | 1122.59 |
|  | Eukaryota | 2022 | Summer | 28.38% |  | 61.11% | 0.41% | 10.09% | 327.27 |
|  |  | 2023 | Spring | 18.02% |  | 73.98% | 0.39% | 7.62% | 428.66 |
|  |  | 2023 | Summer | 17.4% |  | 64.64% | 0.91% | 17.05% | 299.14 |
